# Prevalence of obstructive sleep apnea after treatment for advanced T-stage head and neck cancer

**DOI:** 10.1007/s00405-024-08467-6

**Published:** 2024-02-07

**Authors:** R. T. Karsten, J. A. Rijken, I. Toprak, E. Kant, R. de Bree, L. E. Smeele, M. W. M. van den Brekel, N. de Vries, M. J. L. Ravesloot

**Affiliations:** 1 Department of Head and Neck Oncology and Surgery, The Netherlands Cancer Institute, Amsterdam, The Netherlands; 2 Department of Head and Neck Surgical Oncology, University Medical Center Utrecht, Utrecht, The Netherlands; 3 Amsterdam Center for Language and Communication/ACLC-Institute of Phonetic Sciences, University of Amsterdam, Amsterdam, The Netherlands; 4 Department of Oral and Maxillofacial Surgery, Amsterdam University Medical Center, Amsterdam, The Netherlands; 5 Department of Otorhinolaryngology and Head and Neck Surgery, OLVG, Amsterdam, The Netherlands; 6 Plesmanlaan 121, 1066 CX Amsterdam, The Netherlands

**Keywords:** Obstructive sleep apnea, Head and neck cancer, (Chemo)radiotherapy, Surgery, Posttreatment, Survivors

## Abstract

**Purpose:**

Treatment of head and neck cancer (HNC) may lead to obstructive sleep apnea (OSA), but conclusive results on the prevalence of OSA are lacking. The objective of this study is to investigate the prevalence of OSA in a cohort of patients treated for advanced T-stage HNC.

**Methods:**

A cross-sectional study was conducted in two tertiary cancer care centers including patients at least 1 year after treatment with curative intent with surgery and/or (chemo)radiotherapy ((C)RT) for advanced T-staged (T3-4) cancer of the oral cavity, oropharynx, hypopharynx, or larynx. A polysomnography (PSG) was performed in all participants. OSA was defined as an apnea–hypopnea index (AHI) of 15 events/h or higher or an AHI of 5 events/h and higher with OSA related symptoms, such as sleeping problems, daytime dysfunction and/or cardiac/metabolic comorbidities collected through file review and questionnaires.

**Results:**

Of the 67 participants, 48 (72%, 95% CI 59–82%) were diagnosed with OSA. Possible risk factors are male gender, higher BMI, greater neck circumference, more nicotine pack years, cardiometabolic comorbidities, use of medication with sleepiness as side effect, present tonsils, lower T-stage (T3 vs. T4 stage), higher AJCC stage and a HPV-negative tumor.

**Conclusion:**

In this population of advanced T-stage HNC patients, the prevalence of OSA was 72%, which is considerably higher than in the general population (2–50%). Given the high prevalence, screening of this entire subgroup for OSA may be indicated. Future studies to identify high risk factors and develop an OSA screening protocol are needed.

## Introduction

Obstructive sleep apnea (OSA) is characterized by complete or partial obstruction of the upper airway during sleep, resulting in oxygen desaturations [1]. Being the most common sleep disorder, the prevalence in the general population lies between 2–23% and 4–50% for women and men respectively, depending on gender, age, and definition of the criteria used [2–5]. OSA is associated with increased morbidity and mortality, symptoms of excessive daytime sleepiness and insomnia, resulting in reduced quality of life (QoL) and an increased risk of traffic accidents and physical conditions such as cardiovascular complications and even cancer [5–9].

Treatment of head and neck cancer (HNC) can affect the anatomy of the upper airway and upper digestive tract, often resulting in impairment of speech and swallowing function [10, 11]. Another consequence of this altered anatomy is that it may lead to OSA [12, 13]. Early recognition and treatment of OSA in HNC survivors may contribute to an improvement of their QoL.

Several studies have been conducted to determine the prevalence of OSA after treatment for HNC [12, 13]. Their results, however, vary tremendously with prevalence rates ranging from 12 to 96% [13–23]. This large variation can partially be explained by difference of the patient cohorts regarding tumor site, disease stage and treatment strategies as well as by the varying definitions of OSA used. To date, conclusive results on the prevalence of OSA among HNC patients and clear insights into high-risk subgroups are lacking.

The objective of this study is to investigate the prevalence of OSA in a cohort of patients treated for advanced T-stage HNC. In case OSA is indeed a relevant disorder among these patients, more attention should be given to improve doctors’ awareness and to develop screening programs.

## Methods

### Trial design

A cross-sectional study was conducted on the prevalence of OSA in advanced T-stage HNC patients after treatment. The Medical Ethical Committee (MEC) of the Netherlands Cancer Institute Antoni van Leeuwenhoek (NKI-AVL) granted approval for the study (METC19.1819/M17OSA). The University Medical Center Utrecht (UMCU) complies with these MEC requirements. Written informed consent was obtained from all participants.

### Participants

Patients who visited the department of Head and Neck Oncology and Surgery of the NKI-AVL or department of Head and Neck Surgical Oncology of the UMCU from October 2019 till February 2021 and were at least one year after treatment with surgery and/or (chemo)radiotherapy ((C)RT) with curative intent for advanced T-staged (T3-4) cancer of the oral cavity, oropharynx, hypopharynx, or larynx were asked to participate in this study. Exclusion criteria included a tracheostoma, recurrent disease or distant metastasis. Inclusion took place at the outpatient clinic and all eligible patients visiting the outpatient clinic were asked to participate (on the days the first, third or fourth author were available). In case a patient refused to participate (non-participant), the following characteristics were recorded with their consent: gender, age, BMI, tumor subsite, TNM-classification (7th edition) [24], human papillomavirus (HPV) status, time since treatment and received cancer treatment.

### Study settings

The study was conducted at the department of Head and Neck Oncology and Surgery of the NKI-AVL and the department of Head and Neck Surgical Oncology of UMCU. The NKI-AVL is a tertiary care center for cancer patients located in Amsterdam, The Netherlands. The UMCU is a tertiary, university medical center located in Utrecht, The Netherlands.

### Data collection

Participant, tumor and treatment characteristics were registered through medical file review. The following characteristics were collected: gender, age, intoxications (tobacco, alcohol, and drug use), cardiovascular comorbidity (e.g., hypertension and diabetes), medication use with sleepiness or a sleep disorder as potential side effect in > 1% of the patients using the medication according to the Farmacotherapeutisch Kompas [25], tumor site, TNM-classification (7th edition), American Joint Committee on Cancer (AJCC) stage, tumor Human Papillomavirus (HPV) status and (time since) cancer treatment. Blood results regarding anemia (Hemoglobine—Hb) and thyroid functions (thyroid stimulating hormone (TSH) and free thyroxine (FT4) levels) were collected from the medical files. The last result since the date of inclusion was used. Anemia was diagnosed in case of Hb levels below 7.5 mmol/L for female participants and below 8.5 mmol/L for male participants. TSH levels below 0.5 mIU/L were considered as hyperthyroidism and above 4.2 mIU/L as hypothyroidism. Cases of hypo- or hyperthyroidism with FT4 levels between 10 and 25 pmol/L were considered subclinical.

Symptom specific questions were asked regarding regular excessive daytime sleepiness, choked breathing during sleep, snoring, sudden awakening from sleep, waking up not feeling rested, lack of concentration, nycturia and (social and/or professional) daytime dysfunction.

The self-reported health and quality of life was assessed by means of the EuroQol-visual analogue scale (EQ-VAS) and European Organization for Research and Treatment of Cancer Quality of Life Questionnaire (EORTC QLQ-C30). The EQ-VAS records the participant’s self-rated health on a visual analog scale ranging from worst (0) to best (100) health [26]. The EORTC QLQ-C30 is a cancer-specific self-report questionnaire and comprises a global quality of life scale (two items), five functional scales, three symptom scales and six single items [27]. The following scales were analyzed for this study: global health status/quality of life, physical functioning, emotional functioning, cognitive functioning, social functioning, fatigue, insomnia. Scores of the QLQ-C30 are linearly transformed to a 0–100 scale (version 3.0), with a higher score indicating a higher level of functioning or global QOL, or a higher level of symptoms or problems.

Physical examination was performed to assess Body Mass Index (BMI), neck circumference (centimeter), tonsil size according to the Friedman classification (0 = absent tonsils, 1 = tonsils hidden within the pillars, 2 = tonsils extending to the pillars, 3 = tonsils extending beyond the pillars but not to the midline, 4 = tonsils extending to the midline) and tongue size according to the modified Mallampati classification (1 = soft palate, fauces, uvula, pillars visible, 2 = soft palate, fauces, uvula visible, 3 = soft palate, base of uvula visible, 4 = soft palate not visible) [28, 29]. During the assessment, the participant was seated in an upright position with mouth opened to a maximum and for the tongue size assessment with their tongue protruded maximally [29].

A full-night home polysomnography (PSG) was performed using a digital polygraph system (outpatient, type 2 PSG). Electroencephalogram (Fp2-C4/Fp1-C3), electrooculogram and submental electromyogram were used to record the sleep pattern. Nasal airflow was measured by a pressure sensor. Thoracoabdominal excursions were registered by straps containing piezoelectric transducers. Pulse oximetry was used to monitor oxygen saturation (SaO2) and heart rate. In addition, movements of the limbs and intensity of snoring were recorded. The following day, data were downloaded to the computer and analyzed by dedicated sleep software. The data was manually reviewed by an experienced neurophysiologist for final analysis. The PSG was scored according to the guidelines of the American Academy of Sleep Medicine [30].

OSA was defined as an AHI of 15 events/h or higher or an AHI of 5 events/h and higher with OSA related symptoms, such as sleeping problems, daytime dysfunction and/or cardiac/metabolic comorbidities according to the criteria of the American Academy of Sleep Medicine (AASM) International Classification of Sleep Disorder third edition (ICSD-3) [31]. In case of OSA diagnosis, the participant was referred for OSA treatment.

### Statistical analysis

Analyses were performed using IBM® SPSS® Statistics 28. Descriptive statistics were used to present medians and ranges for continuous variables and percentages for categorical variables. The 95% confidence interval (CI) was presented for the prevalence of OSA in this sample. Variables between participants versus non-participants were compared using the Mann–Whitney *U* test for continuous variables, the Fisher’s exact test for categorical outcomes or the linear-by-linear approximation of the Chi-square test for ordinal outcomes. Variables between participants with and without OSA were compared using univariable logistic regression analysis. Odds ratios (OR) with corresponding 95% confidence intervals (95% CI) and *p* values were presented.

## Results

### Participant characteristics

Of 116 participants who were eligible and approached at the outpatient clinic, 67 (58%) were willing to participate. Characteristics of participants and non-participants are presented in Table 1. In comparison to non- participants, participants were more likely to have had a tumor of the oral cavity (31% vs. 12%, *p* = 0.055) or stage IV disease (76% vs 57%, *p* = 0.043) compared to non-participants.

**Table 1:** Characteristics of participants and non-participants

	Participants (*n* = 67) *N* (%)	Non-participants (*n* = 49) *N* (%)	*P* value
Gender			
Male	44 (66)	29 (59)	0.560^b^
Female	23 (34)	20 (41)	
Age (years) Median (range)	66 (37–89)	66 (44–89)	0.810^a^
BMI (kg/m^2^) Median (range)	24 (17–47)	22 (17–33)	0.147^a^
Tumor site			
Oral cavity	21 (31)	6 (12)	0.055^b^
Oropharynx	24 (36)	23 (47)	
Larynx	19 (28)	14 (29)	
Hypopharynx	3 (5)	6 (12)	
T-stage			
T3	41 (61)	34 (69)	0.433^c^
T4	26 (39)	15 (31)	
N-stage			
N0	27 (40)	20 (41)	1.000^c^
N1	10 (15)	5 (10)	
N2	27 (40)	23 (47)	
N3	3 (5)	1 (2)	
AJCC-stage			
III	16 (24)	21 (43)	**0.043**^**c**^
IV	51 (76)	28 (57)	
HPV status			
Negative	17 (61)	15 (63)	1.000^b^
Positive	11 (39)	9 (38)	
Unknown	39	25	
Received treatment			
Surgery	7 (10)	2 (4)	0.624^b^
RT	18 (27)	16 (33)	
CRT	28 (42)	24 (49)	
Surgery + (C)RT	14 (21)	7 (14)	
Unknown	0	6	
Months since treatment Median (range)	30 (12–272)	27 (12–255)	0.769^a^

*P* values of either the Mann–Whitney U test^a^ or Fisher’s exact test^b^. or linear-by-linear approximation of the Chi-square test^c^. Boldfaced *p* values are statistically significant

Abbreviations: *AJCC* American Joint Committee on Cancer, *BMI* body mass index, *(C)RT* (chemo)radiotherapy, *HPV* human papilloma virus

### PSG and symptom specific questions

PSG results and answers to symptom specific questions are presented in Table 2. Forty-eight participants (72%, 95% CI 59–82%) were diagnosed with OSA. The median AHI was 9 events/h (range 0–93). Nineteen participants (28%) had an AHI ≤ 5 events/h, 27 (40%) of 6–15 events/h, 13 (19%) of 16–30 events/h and 8 (12%) > 30 events/h. The median time spent with an oxygen saturation below 90% during the sleep test was 1 min (range 0–322 min). Median percentage of snoring during the total sleeping time was 18% (range 0–91). Thirty-four participants (51%) suffered from daytime sleepiness, 7 (10%) of choked breathing during sleep, 40 (60%) reported snoring, 16 (24%) regularly woke up suddenly during sleep, 22 (33%) regularly woke up tired, 18 (27%) suffered from lack of concentration, 31 (46%) had nycturia and 12 (18%) reported daytime (social and/or professional) dysfunction. Seven participants (10%) had none of the previously mentioned symptoms. Forty-four participants (66%) had cardiometabolic comorbidities.

**Table 2:** Polysomnography and symptom specific questions

	*N* (%)
Polysomnography	
AHI *events/hour* Median (range)	9 (0–93)
AHI *events/hour*	
0–< 5	19 (28)
≥ 5–< 15	27 (40)
≥ 15–< 30	13 (19)
≥ 30	8 (12)
Minutes of O_2_ saturation < 90% Median (range)	1 (0–322)
% time snoring during sleep Median (range)	18 (0–91)
Symptom specific questions	
Do you often experience daytime sleepiness?	
No	33 (49)
Yes	34 (51)
Do you have choked breathing during sleep?	
No	60 (90)
Yes	7 (10)
Do you snore?	
No	27 (40)
Yes	40 (60)
Do you experience repeated sudden wakening during sleep?	
No	51 (76)
Yes	16 (24)
Are you tired when waking up?	
No	45 (67)
Yes	22 (33)
Do you experience a lack of concentration?	
No	49 (73)
Yes	18 (27)
Do you often have to go to the toilet during the night?	
No	36 (53)
Yes	31 (46)
Do you have problems with daytime functions (social/work related)?	
No	55 (82)
Yes	12 (18)
Number of symptoms*	
0	7 (10)
1	14 (21)
2	14 (21)
3	12 (18)
4	6 (9)
5	8 (12)
6	5 (8)
7	0 (0)
8	1 (2)
Comorbidities	
Cardiometabolic comorbidities**	
No	23 (34)
Yes	44 (66)
Conclusion	
OSA	
No	19 (28)
Yes	48 (72)

Abbreviations: *AHI* Apnea–Hypopnea Index, *OSA* obstructive sleep apnea defined as an AHI ≥ 15 events/hour or an AHI ≥ 5 events/hour with sleeping problems, daytime dysfunction or cardiac and/or metabolic comorbidities

^*^Number of symptoms from the symptom specific questions

^**^Cardiometabolic comorbidities included hypertension, diabetes, aortic aneurysm, intermittent claudication, pulmonary embolism, (cardial) vascular disease (including myocardial infarction), heart failure, cardiac arrythmia, hypercholesterolemia, cerebrovascular accident

### Self-reported health and quality of life

Self-reported health and QoL of participants with and without OSA are listed in Table 3. Self-reported health based on the median EQ-VAS was comparable between participants with and without OSA (80 vs. 80, *p* = 0.353). Although not statistically significant, participants with OSA had a slightly lower global health status (QoL according to EORTC-QLQ-C30 subscale; 75 vs. 83, *p* = 0.657). All other subscales were comparable except for the fatigue subscale, which showed a trend for higher scores in the OSA group (22 vs. 0, *p* = 0.087) indicating more symptoms of fatigue.

**Table 3:** Self-reported health and quality of life of participants with and without OSA

	OSA (*n* = 48*) *N* (%)	No OSA (*n* = 19*) *N* (%)	*p* value
EQ-VAS			
EQ-VAS Median (range)	80 (30–100)	80 (60–98)	0.353
EORTC-QLQ-C30			
Global health status/QoL Median (range)	75 (42–100)	83 (42–100)	0.657
Physical functioning Median (range)	87 (0–100)	93 (40–100)	0.145
Role functioning Median (range)	100 (0–100)	100 (33–100)	0.438
Emotional functioning Median (range)	92 (33–100)	92 (50–100)	0.766
Cognitive functioning Median (range)	100 (33–100)	100 (67–100)	0.458
Social functioning Median (range)	100 (17–100)	100 (17–100	0.877
Fatigue Median (range)	22 (0–89)	0 (0–67)	0.087
Insomnia Median (range)	0 (0–100)	0 (0–100)	0.573

*P* values of the Mann–Whitney *U* test. Boldfaced *p* values are statistically significant

Abbreviations: *EORTC-QLQ-C30* European Organization for Research and Treatment of Cancer Quality of Life Questionnaire, *EQ-VAS* EuroQol-visual analogue scale, *OSA* obstructive sleep apnea

^*^43 of the 48 participants (90%) participants with OSA and 17 of the 19 participants (89%) without OSA filled out the questionnaires

### Participants with vs. without OSA

In Table 4 differences in variables between participants with and without OSA are presented. In this study, the participants with OSA were more often male (52% vs. 27%, OR 0.3 [95% CI 0.1–1.0], *p* = 0.051), had a higher median BMI (25 vs. 22 kg/m^2^, OR 1.2 [95% CI 1.0–1.5], *p* = 0.020), had a greater neck circumference (39 vs. 36 cm, OR 1.3 [95% CI 1.1–1.5], *p* = 0.006), were more often current smokers (33% vs. 21%, OR 1.6 [95% CI 0.2–11.5], *p* = 0.640), had smoked more pack years (36 vs. 20, OR 1.0 [95% CI 1.0–1.0], *p* = 0.617), were more likely to be using medication with potential sleepiness as a side effect (56% vs. 32%, OR 2.8 [95% CI 0.9–8.6], *p* = 0.074), and were more likely to still have their tonsils (66% vs. 47%, OR 2.2 [95% CI 0.7–6.6], *p* = 0.152). There was no clear trend in Mallampati classifications between participants with and without OSA. Concerning the head and neck malignancies, patients with OSA were less likely to have had tumors located in the oral cavity (29% vs. 37%, *p* = 0.852) and were more likely to have had a tumor in the larynx (31% vs. 21%, OR 1.9 [95% CI 0.5–7.8], *p* = 0.388), less often a T4 tumor (31% vs. 58%, OR 0.3 [95% CI 0.1–1.0], *p* = 0.048), a higher AJCC-stage (79% vs. 68% stage IV, OR 1.8 [95% CI 0.5–5.8], *p* = 0.356), and their tumors were less often HPV-positive (35% vs. 50%, OR 0.5 [95% CI 0.1–2.8], *p* = 0.466). Participants with OSA more often reported symptoms included in the symptom specific questions including daytime sleepiness (58% vs. 32%, OR 3.0 [1.0–9.3), *p* = 0.053], snoring 67% vs. 42%, OR 2,8 [0.9–8.2], *p* = 0.0,69 and sudden wakening 27% vs. 16%, OR 2.0 [0.5–7.9], *p* = 0.334).

**Table 4:** Characteristics between participants with and without OSA statistically compared with univariable analysis presented in odds ratios and *p* values

	Number of participants				Univariable analysis	
	OSA (*n* = 48)	No OSA (*n* = 19)	Total (*n* = 67)		OR (95%CI)	*p* value
Participant characteristics						
Gender						0.051
Male	35 (73)	9 (47)	44 (66)	Male	1.0	
Female	13 (27)	10 (52)	23 (34)	Female	0.3 (0.1–1.0)	
Age (years) *Median (range)*	66 (44–86)	67 (37–89)	66 (37–89)		1.0 (1.0–1.1)	0.540
BMI (kg/m^2^) Median (range)	25 (17–47)	22 (17–30)	24 (17–47)		1.2 (1.0–1.5)	**0.020**
Neck circumference (cm) *Median (range)*	39 (29–52)	36 (28–42)	38 (28–52)		1.3 (1.1–1.5)	**0.006**
Smoking						0.604
Never	5 (10)	2 (11)	7 (10)	Never	1.0	
Stopped	27 (56)	13 (68)	40 (60)	Stopped	0.8 (0.1–4.9)	0.837
Current	16 (33)	4 (21)	20 (30)	Current	1.6 (0.2–11.5)	0.640
Pack years *Median (range)*	36 (0–68)	20 (0–68)	32 (0–68)		1.0 (1.0–1.0)	0.617
Units alcohol/week *Median (range)*	2 (0–35)	1 (0–28)	2 (0–35)		1.0 (1.0–1.1)	0.261
Drug use						
No	48 (100)	18 (95)	66 (99)	No	NA	
Yes	0 (0)	1 (5)	1 (2)	Yes		
Cardiometabolic comorbidities*						0.051
No	13 (27)	10 (53)	23 (34)	No	1.0	
Yes	35 (73)	9 (47)	44 (66)	Yes	3.0 (1.0–9.0)	
Hypertension						**0.014**
No	26 (54)	17 (90)	43 (64)	No	1.0	
Yes	22 (46)	2 (11)	24 (36)	Yes	7.2 (1.5–34.6)	
Diabetes						0.203
No	36 (75)	17 (90)	53 (79)	No	1.0	
Yes	12 (25)	2 (11)	14 (21)	Yes	2.8 (0.6–14.1)	
Medication with sleepiness as side effect						0.074
No	21 (44)	13 (68)	34 (51)	No	1.0	
Yes	27 (56)	6 (32)	33 (49)	Yes	2.8 (0.9–8.6)	
Tonsil size (Friedman classification)						0.152
0	15 (33)	10 (53)	25 (39)	0	1.0	
1	28 (62)	8 (42)	36 (56)	1–4	2.2 (0.7–6.6)	
2	2 (4)	1 (5)	3 (5)			
3	0 (0)	0 (0)	0 (0)			
4	0 (0)	0 (0)	0 (0)			
Unknown	3	0	3			
Tongue size (Mallampati classification)						0.356
1	16 (36)	5 (26)	21 (33)	1	1.0	
2	5 (11)	4 (21)	9 (14)	2	0.4 (0.1–2.0)	0.265
3	16 (36)	4 (21)	20 (31)	3	1.3 (0.3–5.8)	0.769
4	8 (18)	6 (32)	14 (22)	4	0.4 (0.1–1.8)	0.240
Unknown	3	0	3			
Tumor and treatment characteristics						
Tumor site						0.852
Oral cavity	14 (29)	7 (37)	21 (31)	Oral cavity	1.0	
Oropharynx	17 (35)	7 (37)	24 (36)	Oropharynx	1.2 (0.3–4.3)	0.763
Larynx	15 (31)	4 (21)	19 (28)	Larynx	1.9 (0.5–7.8)	0.388
Hypopharynx	2 (4)	1 (5)	3 (5)	Hypopharynx	1.0 (0.1–13.0)	1.000
T-stage						**0.048**
T3	33 (69)	8 (42)	41 (61)	T3	1.0	
T4	15 (31)	11 (58)	26 (39)	T4	0.3 (0.1–1.0)	
N-stage						0.406
N0	18 (38)	9 (47)	26 (40)	N0	1.0	
N1	8 (17)	2 (11)	10 (15)	N1	2.0 (0.4–11.4)	0.436
N2	21 (44)	6 (32)	27 (40)	N2	1.8 (0.5–5.9)	0.365
N3	1 (2)	2 (11)	3 (5)	N3	0.3 (0.0–3.1)	0.283
AJCC-stage						0.356
III	10 (21)	6 (32)	16 (24)	III	1.0	
IV	38 (79)	13 (68)	51 (76)	IV	1.8 (0.5–5.8)	
HPV status						0.466
Negative	13 (65)	4 (50)	17 (61)	Negative	1.0	
Positive	7 (35)	4 (50)	11 (39)	Positive	0.5 (0.1–2.8)	
Unknown	28	11	28			
Received treatment						0.913
Surgery	5 (10)	2 (11)	7 (10)	Surgery	1.0	
RT	14 (29)	4 (21)	18 (27)	RT	1.4 (0.2–10.1)	0.739
CRT	19 (40)	9 (47)	28 (42)	CRT	0.8 (0.1–5.2)	0.859
Surgery and (C)RT	10 (21)	4 (21)	14 (21)	Surgery and (C)RT	1.0 (0.1–7.5)	1.000
Months since treatment Median (range)	28 (12–272)	30 (12–197)	30 (12–272)		1.0 (1.0–1.0)	0.872
Blood characteristics						
Anemia						0.830
No	25 (56)	10 (53)	35 (55)	No	1.0	
Yes	20 (44)	9 (47)	29 (45)	Yes	0.9 (0.3–2.6)	
Unknown	3	0	3			
Thyroid function						0.463
Euthyroidism	27 (84)	10 (71)	37 (81)	Euthyroidism	1.0	
Subclinical Hypothyroidism	5 (16)	4 (29)	9 (20)	Subclinical hypothyroidism	0.5 (0.1–2.1)	
Hypothyroidism	0 (0)	0 (0)	0 (0)			
Unknown	16	5	21			
Symptom specific questions						
Daytime sleepiness						0.053
No	20 (42)	13 (68)	33 (49)	No	1.0	
Yes	28 (58)	6 (32)	34 (51)	Yes	3.0 (1.0–9.3)	
Choked breathing						0.989
No	43 (90)	17 (90)	60 (90)	No	1.0	
Yes	5 (10)	2 (11)	7 (10)	Yes	1.0 (0.2–5.6)	
Snoring						0.069
No	16 (33)	11 (58)	27 (40)	No	1.0	
Yes	32 (67)	8 (42)	40 (60)	Yes	2.8 (0.9–8.2)	
Sudden wakening						0.334
No	35 (73)	16 (84)	51 (76)	No	1.0	
Yes	13 (27)	3 (16)	16 (24)	Yes	2.0 (0.5–7.9)	
Waking up not rested						0.476
No	31 (65)	14 (74)	45 (67)	No	1.0	
Yes	17 (35)	5 (26)	22 (33)	Yes	1.5 (0.5–5.0)	
Lack of concentration						0.585
No	36 (75)	13 (68)	49 (73)	No	1.0	
Yes	12 (25)	6 (32)	18 (27)	Yes	0.7 (0.2–2.3)	
Nycturia						0.512
No	27 (56)	9 (47)	36 (53)	No	1.0	
Yes	21 (44)	10 (53)	31 (46)	Yes	0.7 (0.2–2.0)	
Daytime dysfunction						0.674
No	40 (83)	15 (79)	55 (82)	No	1.0	
Yes	8 (17)	4 (21)	12 (18)	Yes	0.8 (1.2–2.9)	
Number of symptoms**						0.285
0	4 (8)	3 (16)	7 (10)	0	1.0	
1	10 (21)	4 (21)	14 (21)	1	1.9 (0.3–12.5)	0.515
2	10 (21)	4 (21)	14 (21)	2	1.9 (0.3–12.5)	0.515
3	10 (21)	2 (11)	12 (18)	3	3.8 (0.4–31.6)	0.224
4	2 (4)	4 (21)	6 (9)	4	0.4 (0.0–3.6)	0.396
5	6 (13)	2 (11)	8 (12)	5–8	4.5 (0.5–37.4)	0.164
6	5 (10)	0 (0)	5 (8)			
7	0 (0)	0 (0)	0 (0)			
8	1 (2)	0 (0)	1 (2)			
Number of symptoms**						0.376
0	4 (8)	3 (16)	7 (10)	0	1.0	
1–8	44 (92)	16 (84)	60 (90)	1–8	2.0 (0.4–10.2)	

Boldfaced *p* values are statistically significant

Abbreviations: *BMI* body mass index, *(C)RT* (chemo)radiotherapy, *HPV* human papilloma virus, *OR* odds ratio, *OSA* obstructive sleep apnea

^*^Metabolic comorbidities included hypertension, diabetes, aortic aneurysm, intermittent claudication, pulmonary embolism, (cardial) vascular disease (including myocardial infarction), heart failure, cardiac arrythmia, hypercholesterolemia, cerebrovascular accident

^**^Number of symptoms from the symptom specific questions

## Discussion

In this cross-sectional study, the prevalence of OSA in 67 advanced T-stage (T3-4) HNC patients at least one year after definitive treatment with curative intent (surgery and/or [C]RT) was 72%, which is considerably higher than in the general population (2–50%). More prevalent variables among the participants with OSA (although not all statistically significant) were male gender, higher BMI, greater neck circumference, more pack years, use of medication with sleepiness as side effect, present tonsils (Friedman classification > 0), T3 stage, higher AJCC stage and an HPV-negative tumor.

The risk for selection bias was kept as small as possible by asking all eligible patients to participate on days inclusion took place. However, 42% of the asked participants were not willing to participate. Baseline characteristics were not fully comparable between participants and non-participants. Participants more often had an oral cavity tumor, less often an oropharyngeal tumor and more often stage IV disease. What the impact of these differences is regarding over- or under-estimation of the prevalence remains unclear because univariable analysis showed less oral cavity tumors in the OSA group, but more stage IV disease. A second factor possibly introducing selection bias is that the cohort was not consecutively recruited. Although we cannot say this for sure since we have no data on patients not approached for participation, patients visiting the outpatient clinic on days when inclusion did not take place are not expected to have a different risk for OSA than patients who were approached. A third factor possibly impacting generalizability is that patients with OSA symptoms might be more likely to participate than patients without, inducing overestimation of the actual prevalence. Sensitivity analysis showed that in case none of the non-participants had OSA, the prevalence would still be 41%. Also, we believe that patients who were not willing to participate in this study possibly would also not be willing to participate in screening by means of PSG. Therefore, we think that these results are representative for the group of advanced T-stage HNC patients that are open to screening.

Several research groups have investigated OSA prevalence in HNC populations [12, 13, 15–17, 19–23, 32–34]. The estimated prevalence in these studies, however, varied greatly, from 12 to 96% [15, 19]. This variation may be caused by several factors. First, patient samples vary regarding tumor site, stage, and treatment modality. Second, the researchers used different AHI cut-off values and definitions for OSA. Third, not all participants of these studies underwent a PSG. In the study by Nesse et al., who found an OSA prevalence of 12% in their sample, only participants with complaints were offered to undergo a PSG which might have caused underestimation of the prevalence [15]. Fourth, small sample sizes may have caused atypical sampling. To our knowledge, only three studies on this subject have included over 50 participants [17, 22, 33], of which only the one performed by Loth et al. was a prospective study with PSG registration in all participants [17]. In this study, 51 participants were included with stage III-IV oropharyngeal cancer treated with CRT or surgery combined with (C)RT. Thirteen participants (25%) had an AHI greater than 10 events/hour. Another study with a large sample was that of Gavidia et al. [22]. They included 67 patients with HNC at least one year after treatment but did not perform PSG in all participants. Only the risk for OSA was determined by means of a questionnaire (STOP-BANG) and they found that 60% of the participants had an elevated OSA risk. Nevertheless, like our findings, most studies found a high prevalence of OSA suggesting that the HNC population is at higher risk.

In this study, we analyzed possible risk factors for OSA by performing univariable analyses. The reason HPV-negative tumors were associated with a slightly higher risk (not statistically significant) for OSA might be that these patients are often older, have smoked more pack years and are less fit than patients with an HPV-positive tumor and therefore damage of tumor and treatment on the anatomy of the upper airway might be less reversible [35]. Oddly, lower T-stage (T3 vs. T4) was associated with a higher risk for OSA in this sample. It is still possible that tumor volume, which might be larger in T3 tumors compared to T4 tumors, is associated with a higher risk for OSA which causes more tissue damage after treatment. The Friedman and Mallampati classifications, which are associated with the severity of OSA in the general population, were not associated with OSA in this sample [36]. This might be a hint that OSA in HNC patients is not caused by obstruction of the tongue or tonsils but other, more caudally located, obstructions may play a more important role in the pathophysiology. Fibrosis and lymphedema might play a role in the etiology. We must state that due to the relative heterogeneity of this cohort (a relatively small sample size of participants with the same tumor site and treatment modality received), we should interpret these results with some caution.

Other studies on this subject show varying results regarding risk factors for OSA [13]. Again, this is probably due to small size of and heterogeneity between patient samples. Therefore, in our opinion, no conclusions can be drawn on (known) predictors for OSA after HNC treatment. This was one of the reasons we decided not to develop a prediction model to identify high risk patients for targeted screening. In literature, however, some variables were found to be associated with OSA in HNC patients. For example, although not always statistically significant, several studies showed that male gender was more prevalent in patients diagnosed with OSA compared to female gender [15, 16, 33, 34]. This was also the case for higher tumor or disease stage or size [15, 21]. In contrast however, Huppertz et al. found that patients with small cancers had a higher risk for OSA [23]. Other studies found no association [16, 20, 22, 32]. Also, type of tumor treatment, especially the involvement of radiotherapy might be associated with OSA after treatment. However, also because of small sample sizes results were inconclusive [17, 19, 21, 22, 33].

The current study identified a higher prevalence of OSA amongst advanced T-stage HNC patients. The pathophysiology, however, remains unclear and additional studies on this are needed. OSA is caused by an obstruction in the upper airway. Where this obstruction is localized in the treated HNC patients might differ greatly between patients. For example, obstruction due to fibrosis or (lymph)edema might play a role in obstructing the airway at several levels. Also, due to neurogenic disturbances of the larynx, decreased sensation of the pharynx and decreased abduction of the arytenoids can occur. In this study we did not assess whether the participants had OSA prior to the cancer treatment. It could be that patients with a higher risk for OSA prior to treatment were more likely to develop OSA after treatment. Moreover, the prevalence of OSA may increase with age. Future studies should focus on investigating the pathophysiology since this will aid in developing targeted therapies, for example through drug induced sleep endoscopy or imaging, like dynamic magnetic resonance imaging.

Given the high prevalence of OSA in our study population, awareness amongst their treating doctors is crucial to counsel patients and refer treated advanced T-stage HNC patients for PSG and OSA treatment if needed. It would be useful to be able to identify high risk subgroups in the consulting room for targeted screening for OSA with PSG. In fact, the prevalence of OSA in this advanced T-stage HNC patients is such that screening this entire subgroup can be defended. Therefore, we advise to screen all treated patients with advanced T-stage HNC at least 1 year after treatment with PSG and a questionnaire on symptoms. This study gives not enough clues to further select within this high-risk group. We considered only screening patients with symptoms of daytime sleepiness. However, of the 33 participants of this study without daytime sleepiness 20 (61%) had OSA. Future studies to identify high risk factors are needed.

## Conclusion

In this study the prevalence of OSA in advanced T-stage (T3-4) HNC patients at least 1 year after definitive treatment with curative intent was 72%, which is considerably higher than in the general population (2–50%). Given the high prevalence, and the fact that symptoms did not correlate with OSA severity, screening this entire subgroup for OSA may be indicated.

## Data Availability

Not applicable.
